# Peripheral vascular dysfunction in migraine: a review

**DOI:** 10.1186/1129-2377-14-80

**Published:** 2013-10-01

**Authors:** Simona Sacco, Patrizia Ripa, Davide Grassi, Francesca Pistoia, Raffaele Ornello, Antonio Carolei, Tobias Kurth

**Affiliations:** 1 Department of Neurology and Regional Headache Center, University of L’Aquila, Piazzale Salvatore Tommasi 1, L’Aquila, 67100, Italy; 2 Department of Life, Health, and Eviromental Sciences, University of L’Aquila, L’Aquila, 67100, Italy; 3 Inserm Research Center for Epidemiology and Biostatistics, Team Neuroepidemiology, Bordeaux, F-33000, France; 4 University of Bordeaux, Bordeaux, F-33000, France

**Keywords:** Migraine, Migraine with aura, Arterial stiffness, Endothelial function, Flow mediated dilation, Pulse wave velocity, Augmentation index, Stroke, Coronary artery disease, Cardiovascular disease, Risk factors

## Abstract

Numerous studies have indicated an increased risk of vascular disease among migraineurs. Alterations in endothelial and arterial function, which predispose to atherosclerosis and cardiovascular diseases, have been suggested as an important link between migraine and vascular disease. However, the available evidence is inconsistent. We aimed to review and summarize the published evidence about the peripheral vascular dysfunction of migraineurs.

We systematically searched in BIOSIS, the Cochrane database, Embase, Google scholar, ISI Web of Science, and Medline to identify articles, published up to April 2013, evaluating the endothelial and arterial function of migraineurs.

Several lines of evidence for vascular dysfunction were reported in migraineurs. Findings regarding endothelial function are particularly controversial since studies variously indicated the presence of endothelial dysfunction in migraineurs, the absence of any difference in endothelial function between migraineurs and non-migraineurs, and even an enhanced endothelial function in migraineurs. Reports on arterial function are more consistent and suggest that functional properties of large arteries are altered in migraineurs.

Peripheral vascular function, particularly arterial function, is a promising non-invasive indicator of the vascular health of subjects with migraine. However, further targeted research is needed to understand whether altered arterial function explains the increased risk of vascular disease among patients with migraine.

## Introduction

Numerous studies have indicated that migraineurs have an increased risk of vascular diseases. The association between migraine and ischemic stroke was the earliest to be recognized [1-3]. Thereafter, migraine has been associated also with myocardial infarction, hemorrhagic stroke, retinal vasculopathy, cardiovascular mortality, incidental brain lesions, including infarct-like lesions, and in some instances also with peripheral artery disease (Table 1) [4-7]. Mechanisms which may explain these associations have been discussed previously [5,8-10]. One of the plausible mechanisms predisposing individuals to atherosclerosis and vascular diseases is endothelial and arterial dysfunction [11]. Consequently, several studies have evaluated the vascular function of migraineurs outside the brain, providing equivocal results. The aim of the present study was to review the published evidence linking peripheral arterial function with migraine.

**Table 1:** Evidence referring to the association between migraine and the risk of vascular disease


Ischemic stroke	
	Numerous studies demonstrating an association with any migraine [e1-e22];
	Definite association with migraine with aura [e1,e3-e4,e7-e8,e11-e12,e15-e16,e19-e21];
	No definite association with migraine without aura [e1,e3,e7,e11-e12,e16,e19-e20];
	Association with migraine with aura confirmed by three meta-analyses [e23-e25].
Transient ischemic attack	
	The risk seems to be increased in migraineurs, although this issue has not been extensively investigated due to a challenging overlap of symptoms with migraine aura [e6,e19].
Hemorrhagic stroke	
	Several studies addressing the topic and providing inconsistent results [e5,e8-e10,e26-e29];
	A meta-analysis of those studies indicating a small but significant association [e30];
	No definite conclusion regarding migraine type.
Cardiac events	
	Two large studies indicating an association with any migraine in men and women and with migraine with aura in women (data not available for men) [e1,e2]; Conflicting results provided by other available studies [e4,e31-e33];
	No association with any migraine in meta-analysis of data but few studies available [e23]; No analysis according to migraine type due to lack of data.
	A recent study reporting an association between migraine (any migraine, migraine with aura and migraine without aura) and myocardial infarction [e4].
Vascular death	
	A meta-analysis and a large study supporting an association with migraine with aura [e23];
	No association with any migraine according to meta-analysis of data [e23].
Other vascular diseases	
	Studies indicating a possible association with any migraine and retinal disease [e34,e35] and peripheral artery disease [e36-e37].
Brain lesions	
	Migraine has been associated with white-matter hyperintensities and infarct-like lesions [e38-e42];
		Association of migraine with white matter hyperintensities confirmed in two meta-analyses [e41,e43]; no definite association with infarct-like lesions [e43]

References are listed in the online supplement.

### Background information about arterial and endothelial function and its measurements

**Figure 1:**
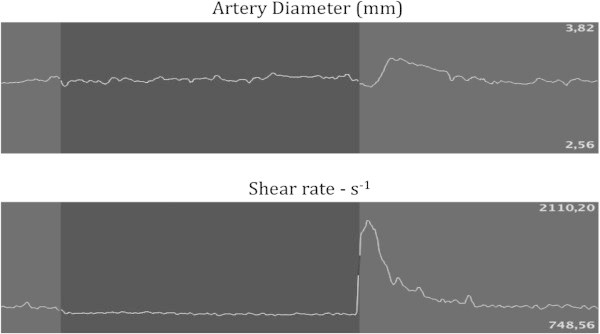
**Flow-mediated dilation.** Flow-mediated dilation (FMD) measures endothelial function by inducing a temporary ischemia in a brachial artery and observing the amount of vasodilation following the stressor event. After a baseline measurement of the artery diameter via an ultrasound probe, a sphygmomanometer blood pressure cuff is positioned on the right forearm 2 cm below the elbow and inflated to 250 mmHg to produce ischemia in the forearm. The cuff is deflated after some minutes, usually 5, thus causing a reactive hyperemia which in turn produces a shear stress stimulus that induces the endothelium to release nitric oxide as a vasodilator. FMD is considered as diameter after reactive hyperemia - basal diameter/basal diameter × 100. The figure shows the diameter (upper part) and shear rate (lower part) of brachial artery during FMD before (grey-shaded part on the left), during (black-shaded part), and after (grey-shaded part on the right) ischemia.

The endothelium, i.e. the inner layer of cells of blood vessels, has physiologically favorable and atheroprotective effects [12]. It works as both a receptor and effector organ and responds to each physical or chemical stimulus with the release of substances, including nitric oxide (NO), with which the endothelium maintains vasomotor balance and vascular-tissue homeostasis [13]. The established cardiovascular risk factors cause oxidative stress initiating a chronic inflammatory process which alters the endothelial cells capacity. This leads to “endothelial dysfunction” with a reduction in endothelium-dependent vasodilation and the induction of a specific state of “endothelial activation,” characterized by a proinflammatory, proliferative, and procoagulatory milieu which favors all stages of atherogenesis, pathological inflammatory processes, and vascular disease [14]. Endothelial dysfunction represents an early step in the development of atherosclerosis [15]. Endothelial function can be investigated by invasive and non-invasive tests [16]. Invasive tests are based upon intra-arterial infusion of vasoactive agents. Among the several non-invasive methods of measuring endothelial function, flow-mediated dilation (FMD), laser Doppler flowmetry, and digital pulse amplitude tonometry (PAT) are the most widely adopted [16-18]. Figure 1 shows details about measurement of FMD [16-19].

**Figure 2:**
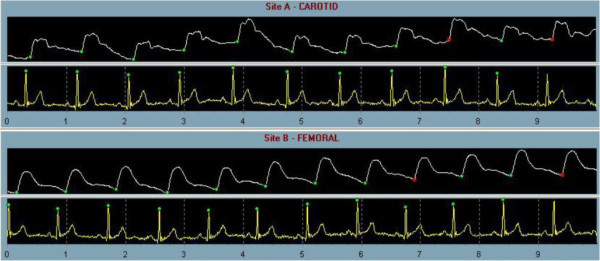
**Carotid-Femoral Pulse Wave Velocity.** Increased arterial stiffness leads to increased velocity of the pulse wave generated in the arteries by the contraction of the left ventricle. Pulse wave velocity (PWV) consists in measuring pulse wave profiles by tonometry at two distant locations (carotid and femoral) and measuring the delay in the onset of the wave between those two locations. PWV is calculated as the distance traveled by the wave divided by the time taken to travel that distance. Surface distance between the two recording sites and simultaneously recorded electrocardiograms are used to calculate wave transit time. The figure shows tonometric (white lines) recordings of the carotid (above) and femoral (below) artery waves according with simultaneous electrocardiographic (yellow lines) ‘R’ wave of the electrocardiogram as a timing reference.

**Figure 3:**
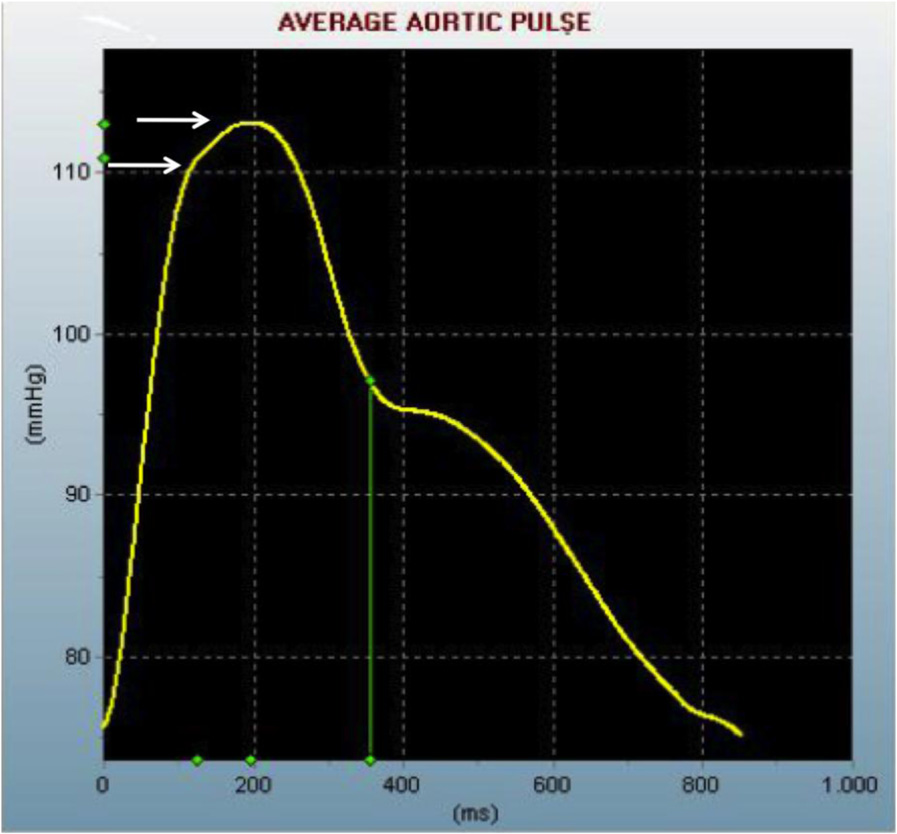
**Augmentation Index.** The interaction between the incident pulse wave and the reflected pulse wave, which is generated by the arterial resistance, is expressed by the augmentation index, which is the amount of pulse wave induced only by arterial resistance. In the figure, the upper arrow indicates the first systolic peak (incident wave), while the lower arrow indicates the second systolic peak (reflected wave). The ratio between the reflected wave/incident wave × 100 is the augmentation index.

Measures of arterial elasticity are also being investigated as non-invasive means of gauging vascular health [14]; the most commonly considered parameters are pulse wave velocity (PWV), augmentation index (AIx) and local arterial distensibility in the carotid artery or in the aorta. While FMD addresses functional status of the endothelium, PWV, AI, and local arterial distensibility are better related to structural changes in the vessel wall. PWV is an indicator of segmental arterial stiffness. Carotid-femoral PWV is the gold standard of assessment (Figure 2), but it is difficult to measure, so in clinical practice it can be replaced by the measurement of brachial-ankle PWV (although this measurement includes some muscular arteries and not only the elastic ones). The AIx (Figure 3) is an index related to the stiffness of the systemic arterial tree. An increase in arterial stiffness leads to a more proximal point of reflection, thus altering the wave profile as measured by the instruments. Local arterial distensibility is assessed by measuring the minimum and maximum diameter of central arteries – such as the carotid artery or aorta – by ultrasound or MRI with simultaneous recording of the blood pressure in the arterial district from which the diameters are obtained.

### Search strategy and selection criteria

Data for this review were obtained through searches in BIOSIS, the Cochrane database, Embase, Google scholar, ISI Web of Science, and Medline from their first availability up to April 2013. The search terms included “migraine” OR “headache” AND (“endothelial function”, “endothelial injury”, “arterial stiffness”, “arterial distensibility”, “vascular resistance”, “vascular reactivity”, “flow-mediated dilation”, “forearm blood flow”, “arterial pulse wave velocity”, “augmentation index”). We also searched reference lists of identified articles and papers quoting identified articles. We reviewed only studies published in English.

## Review

### Arterial and endothelial function in migraineurs

Several lines of evidence for vascular dysfunction have been identified in migraineurs and there is increasing evidence to suggest that in migraineurs the vascular system is impaired not only within the brain.

#### Endothelial function in migraineurs

The many studies assessing endothelial function in migraineurs have used heterogeneous techniques and indicators as shown in Tables 2, 3 and 4. Most of them assessed endothelial function by measuring FMD (Table 2) [20-30], some used PAT (Table 3) [31,32], some measured peripheral vasodilation in response to pharmacological stimuli (Table 3) [20,30,33-35], and others assessed endothelial progenitor cells (EPCs) (Table 4) [23,36,37]. Findings were not consistent across the studies. Several among the available studies did not reveal any alteration in the endothelial function of migraineurs. Six of them did not find any difference in FMD between migraineurs and control subjects (Table 2) [20-23,25,26] and a further study confirmed the lack of any alteration in endothelial function assessed by PAT ratio (Table 3) [32]. Three studies assessing peripheral vasodilation after pharmacological stimuli found comparable responses in subjects with and without migraine (Table 3) [20,33,35]. However, other studies supported the presence of endothelial dysfunction in migraineurs. Four studies reported decreased FMD in migraineurs [24,27,29,30]; in one of those studies, FMD correlated with dysfunction of the autonomic nervous system (Table 2) [24]. Another study, using PAT, reported that subjects with chronic migraine had worse endothelial function (Table 3) [31]. A further study found an abnormal vascular response to dilating pharmacological stimuli (Table 3) [34]. The possible underlying mechanisms of endothelial dysfunction in those studies were unclear. Since the impairment of FMD was demonstrated also in individuals with recent onset of migraine it is unlikely that the disturbance was a consequence of longstanding and repeated exposure to the vasoconstrictor agents used for migraine treatment [27]. In addition, the results of one study suggested that the impaired vascular reactivity in migraineurs is entirely attributable to a reduced response of vascular smooth muscle cells to NO while the endothelial response to NO appeared intact [34]. Some additional studies supported the presence of an endothelial dysfunction in migraineurs by documenting a decreased number of EPCs [23,36] or a higher number of more mature EPCs (which could be associated with potential endothelial damage) in migraineurs (Table 4) [37]. EPCs maintain the integrity of damaged endothelium and are considered a marker of endothelial function [38]. At variance with other studies, Vernieri et al., found that subjects with migraine with aura had higher FMD than both control subjects and migraine without aura subjects indicating an excessive arterial response to hyperemia. It is suggested that this is probably an effect of an increased sensitivity to endothelium-derived NO or of increased release of NO induced by shear stress (Table 2) [28]. In agreement, Yetkin et al., found that migraineurs have an increased nitrate-mediated response showing a major role of NO supersensitivity in migraine pathophysiology (Table 3) [30].

**Table 2:** Studies on endothelial function using flow-mediated dilation in migraineurs

**Study (First author, year)**	**Study design**	**Patients included (n)**	**% women**	**Exclusion criteria**	**Migraine diagnosis**	**Results**
De Hoon, 2006 [20]	CC	Migraine: 10	60	CVD, HYP, DM, dyslipidemia, CS	ICHD-I	No differences in FMD between migraineurs and controls
		Controls: 10				
Hamed, 2010 [21]	CC	Migraine: 38	89	CVD and vascular RF, DM, CS, alcoholism, active gastrointestinal disease, gout, epilepsy, recent infection, renal failure, pregnancy or lactation, regular use FANS or antimigrainous drugs and OC	ICHD-II	No differences in FMD between migraineurs and controls
		(MwA: 14;				
		MwoA: 24)				
		Controls: 35				
Perko, 2011 [22]	CC	MwA: 20	80	CVD, obesity, HYP, dyslipidemia, pregnancy or lactation and regular use of vasoactive drugs	ICHD-II	No differences in FMD between migraineurs and controls
		MwoA: 20				
		Controls: 20				
Rodriguez-Osorio, 2012 [23]	CC	Migraine : 47	98	CAD, inflammatory disease, obesity, HYP, DM, dyslipidemia, CS, infectious disease, severe systemic disease, ovarium pathology, pregnancy or lactation, use of vasoactive drugs	ICHD-II	No differences in FMD between migraineurs and controls
		(MwA:14;				
		MwoA: 33)				
		Controls: 23				
Rossato, 2011 [24]	CC	Migraine: 20	75	Age ≥50, vascular RF, CS, use of vasoactive drugs	Not reported	Decreased FMD in migraineurs
		Controls: 20				
Silva, 2007 [25]	CC	Migraine: 50 (MwA: 25; MwoA: 25)	88	None	ICHD-II	No differences in FMD between migraineurs and controls
		Controls: 25				
Thomsen, 1996 [26]	CC	MwoA: 12	100	Use of any daily drugs	ICHD-I	No differences in FMD between migraineurs and controls
		Controls: 12				
Vanmolkot, 2007 [27]	CC	Migraine: 50	78	Age >50, CVD, HYP, obesity, DM, dyslipidemia, pregnancy or lactation, use of vasoactive drugs (except OC)	Validated questionnaire	Decreased FMD in migraineurs
		Controls: 50				
Vernieri, 2010 [28]	CC	Migraine:21		None	ICHD-II	FMD increased from controls to MwoA to MwA patients
		(MwA: 11; MwoA: 10)				
		Controls: 13				
Yetkin, 2006 [29]	CC	Migraine: 45	80	CAD, HYP, obesity, DM, infectious disease, ovarium pathology	ICHD-I	Decreased FMD in migraineurs
		Controls: 45				
Yetkin, 2007 [30]	CC	Migraine: 24	89	HYP, CAD, DM, infectious disease	ICHD-I	Decreased FMD in migraineurs
		Controls: 26				

*CC* case–control; *MwA* migraine with aura; *MwoA* migraine without aura; *CVD* cardiovascular disease; *HYP* arterial hypertension; *DM* diabetes mellitus; *CS* cigarette smoking; *RF* risk factors; *FANS* non steroid anti-inflammatory drugs; *OC* oral contraceptives; *CAD* coronary artery disease; *ICHD* International classification headache disorders; *FMD* flow-mediated dilation.

**Table 3:** Studies on endothelial function by arterial tonometry or vasodilation in response to pharmacological stimuli in migraineurs

**Study (First author, year)**	**Study design**	**Patients included (n)**	**% women**	**Exclusion criteria**	**Migraine diagnosis**	**Results**
Arterial tonometry						
Jiménez-Caballero, 2013 [31]	CC	CM: 21	71	Age ≥50, CVD, inflammatory disease, obesity, HYP, DM, dyslipidemia, CS, active cancer, ovarium pathology, pregnancy or lactation, regular use of vasoactive drugs	ICHD-II	Smaller RHI in migraineurs
		Controls: 21				
Liman, 2012 [32]	CC	MwA: 29	100	CVD, obesity, HYP, DM, pregnancy, use of drugs (statins, anticoagulants or antiplatelet and intake of triptans within the previous 24 h)	ICHD-II	No differences in PAT ratio between migraineurs and controls
		Controls: 30				
Vasodilation in response to pharmacological stimuli						
De Hoon, 2006 [20]	CC	Migraine: 10	60	CVD, HYP, DM, dyslipidemia, CS	ICHD-I	No differences between migraineurs and controls in vasodilation response to serotonin, sodium nitroprusside, and CGRP
		Controls: 10				
Edvinsson, 2008 [33]	CC	MwoA: 9	78	None	ICHD-I	No differences between migraineurs and controls in vasodilation response after local heating and iontophoretic administration of acetylcholine, sodium nitroprusside, and CGRP
		Controls: 9				
Napoli, 2009 [34]	CC	MwoA: 12	58	HYP, DM, hypercholesterolemia, CVD, CS	ICHD-I	Reduced response to endothelium-dependent vasodilation and production of cGMP in migraineurs; no difference in production of NO
		Controls: 12				
Vanmolkot, 2010 [35]	CC	Migraine:16	75	Age >50, CVD, HYP, obesity, DM, CS, dyslipidemia, pregnancy or lactation, use of vasoactive drugs (except OC)	ICHD-II	No differences between migraineurs and controls to sodium nitroprusside, substance P, and N^G^-monomethyl-L-arginine
		Controls: 16				
Yetkin, 2007 [30]	CC	Migraine: 24	89	HYP, CAD, DM, infectious disease	ICHD-I	Higher nitrate-mediated dilation in migraineurs
		Controls: 26				

*CC* case–control; *CM* chronic migraine; *MwA* migraine with aura; *MwoA* migraine without aura; *CVD* cardiovascular disease; *HYP* arterial hypertension; *DM*,diabtes mellitus; *CS* cigarette smoking; *OC* oral contraceptive; *ICHD* International classification headache disorders; *RHI* reactive hyperhemia index; *PAT* peripheral arterial tonometry; *CGRP* Calcitonin Gene Related Peptide; *cGMP* cyclic guanosine monophosphate; *NO* nitric oxide.

**Table 4:** Studies on endothelial function by endothelial progenitor cells in migraineurs

**Study (First author, year)**	**Study design**	**Patients included (n)**	**% women**	**Exclusion criteria**	**Migraine diagnosis**	**Results**
Lee, 2008 [36]	CC	Migraine: 92	70	CVD, diabetic retinopathy	ICHD-II	Decreased number and migratory capacity and higher senescence levels of endothelial progenitor cells in migraineurs versus controls
		(MwoA: 67; MwA: 25)				
		Controls: 37				
Oterino, 2013 [37]	CC	CM: 51	73	CVD, inflammatory disease, cancer or treatment with antimitogen agents, pregnancy in the last year	ICHD-I	Higher number of activated endothelial progenitor cells in migraineurs
		EM: 48				
		Controls: 35				
Rodriguez-Osorio, 2012 [23]	CC	Migraine : 47	98	CAD, inflammatory disease, obesity, HYP, DM, dyslipidemia, CS, infectious disease, severe systemic disease; ovarium pathology, pregnancy or lactation, use of vasoactive drugs	ICHD-II	Lower endothelial progenitor cell counts in migraineurs
		(MwA:14; MwoA: 33)				
		Controls: 23				

*CC* case–control; *CM* chronic migraine; *EM* episodic migraine; *MwA* migraine with aura; *MwoA* migraine without aura; *CVD* cardiovascular disease; *CAD* coronary artery disease; *HYP* arterial hypertension; *DM* diabetes mellitus; *CS* cigarette smoking; *ICHD* International classification headache disorders.

Several studies support the presence of biochemical changes in the endothelial activation of migraineurs. These biochemical changes may precede structural damage. In particular, alterations in the NO pathway have been described [30,39,40] as well as increased levels of calcitonin gene-related peptide (CGRP), vascular endothelial growth factors (VEGF), von Willebrand factor (vWF) activity, tissue plasminogen activator antigen, C-reactive protein and endothelin-1 [21,23,40]. Recently, a study involving 40 non-hypertensive subjects with migraine without aura randomized to treatment with enalapril 5 mg twice a day for 3 months or to placebo, showed that active treatment was associated with an increase in FMD values [41].

#### Arterial function in migraineurs

Compared with studies on endothelial function, reports on arterial function measured with PWV, AIx, and local arterial distensibility are more consistent and suggest that functional properties of large arteries are altered in migraineurs [27,42-46] (Table 5).

**Table 5:** Studies on arterial function in migraineurs

**Study (First author, year)**	**Study design**	**Patients included (n)**	**% women**	**Exclusion criteria**	**Migraine diagnosis**	**Parameters assessed**	**Results**
De Hoon, 2003 [46]	CC	MwA: 11	76	CVD, inflammatory disease, HYP, DM, dyslipidemia	ICHD-I	Diameter and compliance parameters of brachial, carotid, femoral, and temporal arteries	Smaller diameter and distension of brachial artery and larger right temporal artery diameter in migraineurs; no differences in carotid and femoral arteries
		MwoA: 39					
		Controls: 50					
Ikeda, 2011 [42]	CC	MwA:22	73	CVD, vascular RF	ICHD-II	Brachial PWV	Higher PWV in migraineurs
		MwoA:89					
		Controls:110					
Jiménez-Caballero, 2013 [31]	CC	CM: 21	71	Age ≥50, CVD, inflammatory disease, obesity, HYP, DM, dyslipidemia	ICHD-II	Radial AIx	Higher AIx in migraineurs
		Controls: 21					
Liman, 2012 [32]	CC	MwA: 29	100	CVD, obesity, arterial HYP, DM	ICHD-II	Peripheral AIx	Higher Aix in migraineurs
		Controls: 30					
Nagai, 2007 [45]	PB	Group A:134	93	Stroke	Validated questionnaire	Radial AIx	Higher AIx in migraineurs
		(5% migraineurs)					
		Group B:138					
		(17% migraineurs)					
Schillaci, 2010 [43]	CC	MwA:17	85	CVD, inflammatory disease, HYP, DM, dyslipidemia	ICHD-II	Aortic PWV and aortic AIx	Higher PWV and AIx in migraineurs, especially in MwA
		MwoA: 43					
		Controls:60					
Stam, 2013 [44]	PB	MwA: 123	75	None	ICHD-II	Carotid and femoral PWV	No differences between migraineurs and controls
		MwoA: 167					
		Controls: 542					
Vanmolkot, 2007 [27]	CC	Migraine: 50	78	None	ICHD-I	Diameter and compliance parameters of brachial and femoral arteries, aortic AIx and aortic PWV	Smaller brachial artery diameter and compliance and smaller femoral artery diameter in migraineurs; higher aortic AIx in migraineurs; higher PWV in migraineurs not confirmed after adjustment for age and mean arterial pressure
		Controls: 50					

*CC* case–control; *PB* population-based; *CM* chronic migraine; *MwA*, migraine with aura; *MwoA*, migraine without aura; *CVD*, cardiovascular disease; *HYP*, arterial hypertension; *DM*, diabetes mellitus; *RF*, risk factors; *ICHD*, International classification headache disorders; *AIx*, augmentation index; *PWV*, pulse wave velocity.

Four studies addressed PWV in migraineurs (Table 5) [27,42-44]. Ikeda et al. evaluated peripheral PWV [42] while the other authors assessed central PWV [27,43,44]. Three studies on PWV [27,42,43] found higher values of this parameter in migraineurs with respect to non-migraineurs. However, in one of the studies PWV did not correlate with the presence of migraine after adjustment for age and mean arterial pressure [27]. The inclusion in the study of patients with a short history of migraine and with less use of vasoconstrictive agents may explain the difference [27]. Schillaci et al., also addressed PWV according to migraine type and reported that subjects with migraine either with or without aura had increased PWV with respect to controls while migraine with aura subjects had higher aortic PWV than those without aura [43]. A more recent population-based study, involving a larger number of migraineurs, has challenged the suggestion of possible impairment of arterial function in migraineurs [44]. In fact, Stam et al. reported no differences in PWV among subjects with migraine with aura, migraine without aura, and controls [44]. In this latter study subjects with known cardiovascular risk factors were included and this may explain the differences in the results [44].

Higher values of AIx were found in migraineurs by five studies [27,31,32,43,45], which variously measured AIx in the radial artery [45], in the aorta [27,43] or by finger tonometry [31,32]. One of those studies involved only women with migraine with aura [32] and one involved only subjects with chronic migraine [31]. Schillaci et al. reported increased AI in migraine with and without aura with respect to controls and no differences between the two groups of migraineurs [43]. All the studies about AIx had a case–control design, except for a population-based one [45] which involved cases and controls older than those of the other studies.

Two studies assessed static arterial parameters [27,46]. De Hoon et al. found that migraineurs had larger temporal artery diameter compared with control subjects but a decreased distensibility and buffering capacity of the brachial artery in the absence of significant alterations in the common carotid and femoral arteries [46]. Vanmolkot et al. found that migraineurs had a decreased diameter and compliance of superficial muscular arteries [27].

## Discussion

The results of the systematic review suggest the presence of a peripheral vascular dysfunction in migraineurs. However, while several studies support an alteration of arterial function among subjects with migraine, findings on the endothelial function are less clear.

Endothelial function in migraineurs has sometimes been reported as impaired and sometimes as altered, while some studies found no difference compared with endothelial function in controls. Differences among available studies may be explained by various inclusion/exclusion criteria and the generally small number of participants. Migraine, and particularly migraine with aura, has been associated with an elevated cardiovascular profile. The exclusion of subjects with cardiovascular risk factors from some of the studies may at least partly explain the described differences. Duration of migraine, severity of attacks and the active or inactive state of the disease may also have played a role in the between-study differences in results. To clarify the status of endothelial function in migraineurs further, studies are required that need to involve large number of subjects of properly selected individuals, applying rigorous inclusion and exclusion criteria, and including subjects with a wide range of migraine conditions in terms of frequency, duration, and severity of the attacks. In addition, further studies should also adopt a standardized and reliable methodology to address endothelial function.

Regarding arterial function, most of the available evidence supports greater stiffness or impaired compliance of the arterial system in migraineurs. The mechanisms underlying the association between migraine and altered arterial function are likely to be complex and may involve both structural and functional changes in the arterial wall. Since all the studies had a cross-sectional design, a cause-effect relationship between migraine and increased arterial stiffness cannot be established. However, migraine usually starts in early adulthood, at an age in which arterial wall pathologies are rare. In the general population alteration in arterial function has been associated with the presence of vascular risk factors and is considered a marker of vascular disease associated with risk of future vascular events [47,48]. Since most studies addressing arterial function in migraineurs excluded subjects with comorbid vascular risk factors, other mechanisms can be hypothesized. The possible hypotheses include alterations in vessel wall structure in migraineurs as supported by the presence of impaired serum elastase activity [49], higher sympathetic tone [50,51], use of drugs such as triptans and ergot derivatives, or it may represent a primary alteration linked to the presence of migraine itself [52]. At the moment it is unknown whether the duration of migraine and the frequency and severity of the attacks has an impact on arterial stiffness. Moreover, gender differences may also play a role in determining arterial stiffness in migraineurs with females being more susceptible to changes [43]. In addition, possible clinical implications of arterial dysfunction in migraineurs have to be clarified. In the general population, arterial dysfunction has been linked to an increased risk of vascular disease through an atherothrombotic mechanism [47,48]. However, in migraineurs the evidence of an atherothrombotic mechanism leading to vascular events is not supported and much evidence points against this possibility. [53,54] In fact, intima-media thickness (IMT), which is a surrogate marker of subclinical atherosclerosis has not been consistently linked to migraine status [20,24,27,55,56]. In addition, despite little information being available on the frequency of the different subtypes of ischemic stroke in migraineurs, the available studies do not support an increased atherosclerotic load [57,58]. Also the cardiac events observed in migraineurs did not seem to be attributable to atherothrombosis and alternative mechanisms have been hypothesized [59-61]. Future studies should also clarify whether the vascular dysfunction may represent a marker able to identify those migraineurs who are at higher risk of presenting vascular events and who might be the target of possible preventive strategies.

## Conclusion

The study of systemic vascular function is a promising tool for non-invasively investigating the vascular health of subjects with migraine. At the moment, evidence is too scarce to support the clinical application of the techniques and further research studies are needed to better clarify all the pending issues.

## Competing interests

The authors declare that they have no competing interests.

## Authors’ contributions

All authors of this manuscript (SS, PR, DG, RO, FP, AC, TK) have made substantial contributions to conception and design of the review, have been involved in drafting the manuscript and revising it critically for important intellectual content and have given final approval of the version to be published.
